# Determination of the Reproductive Status of the Female Round Goby *Neogobius melanostomus* (Pallas, 1814) in Southwestern Lake Michigan

**DOI:** 10.3390/ani16131999

**Published:** 2026-06-29

**Authors:** Meghan A. Kline, Piotr Hliwa, Sergiusz J. Czesny

**Affiliations:** 1 Lake Michigan Biological Station, Illinois Natural History Survey, Prairie Research Institute, University of Illinois, 400 17th Street, Zion, IL 60099, USA; 2 Department of Ichthyology and Aquaculture, University of Warmia and Mazury in Olsztyn, Warszawska St. 117, 10-719 Olsztyn, Poland

**Keywords:** round goby, Lake Michigan, batch spawning, thermal regime, histology of ovaries

## Abstract

The successful invasion of the round goby, *Neogobius melanostomus* (Pallas, 1814), in European and North American water ecosystems is assisted by its reproductive strategy, including high fecundity, multiple spawning events over an extended spawning season, and male parental care of offspring. In this study, we investigated female spawning effort and the determination of their reproductive status. The analysis was based on 552 females caught from May to September in Jackson Harbor (JH) and Waukegan Harbor (WH) in southwestern Lake Michigan (USA). The total length of females ranged from 62.6 to 160.0 mm, and the weight was between 3.47 and 59.8 g. Significantly different maximum mean values of the gonadosomatic index (GSI) in both localities were noted in early June (8.62% for JH) and in mid-June (7.82% for WH), associated with differing thermal regimes. Fecundity exhibited a significant linear relationship with the total length of females at both sites (Jackson Harbor, *p* < 0.0001, R^2^ = 0.5861; Waukegan Harbor, *p* < 0.0001, R^2^ = 0.6278) and with the total mass of fish (Jackson Harbor, *p* < 0.0001, R^2^ = 0.6614; Waukegan Harbor, *p* < 0.0001, R^2^ = 0.6327). Histological examination of ovaries showed that the spawning season of the round goby in southwestern Lake Michigan is prolonged from May to late June. The monthly distribution of the different sexual maturity stages and the frequency distribution of oocyte diameter revealed that this species is a batch spawner. The results expand the current knowledge of reproductive biology and physiology of the round goby.

## Simple Summary

The round goby *Neogobius melanostomus* is a species within a large, tolerant, prolific family, Gobiidae, and has successfully invaded the Laurentian Great Lakes over the past four decades. Among the many reasons for their success are physiological factors that allow the round goby to rapidly expand its range. This study evaluated spawning effort and the determination of reproductive status of female round goby in two sites of southwestern Lake Michigan, i.e., Jackson and Waukegan Harbors. Spawning peaks differed between two relatively proximate sites and occurred at 14–17 °C. Peak spawning effort, as determined by gonadosomatic index score, correlates with the highest proportions of mature oocytes and confirms their usefulness as a good indicator of spawning activity. Monthly egg counts were largely unchanged throughout the reproductive season (May–September), confirming continuous development and maturation of oocyte batches during a protracted spawning period. Significant differences in proportional oocyte counts per sample period were present for stage 1 (immature) and stage 3 (mature) oocytes, which aids in the validation of predicted spawning peaks from GSI scores. Fecundity was significantly positively related to the total length and total weight of individual females.

## 1. Introduction

The round goby, *Neogobius melanostomus* (Pallas, 1814), native to the Ponto-Caspian region, is one of the most wide-ranging invasive fish on earth [1]. The distribution of the round goby in Europe has greatly expanded in recent years, with established populations now present along the Baltic Sea coast [2,3,4,5,6], the middle and especially the upper Danube, Rhine, and Vistula river basins [7,8,9,10]. In North America, the round goby was first observed in the St. Clair River in 1990 [11], a waterway that connects Lakes Erie and Huron. In 1993, the round goby was observed in the Calumet River near Lake Michigan [12,13] after a presumed initial introduction via ballast water exchange along international shipping routes within the Great Lakes [14,15]. After its introduction, the round goby quickly spread to all five Great Lakes [16,17,18]. During the last few years, round gobies also colonized large river systems, such as the Illinois River [19], and many small Great Lakes tributaries [20,21,22]. It has also spread through the Great Lakes into wetland and tributary habitats directly by dispersal and into inland lakes indirectly through human-aided movement, e.g., bait-bucket transfer and accidental release [1].

Range expansion of the round goby is promoted by several attributes typical for the successful invaders, such as tolerance of a wide range of environmental factors (e.g., salinity, light, temperature), opportunistic feeding strategy, and aggressive inter- and intra-specific behaviors [23,24,25]. Early sexual maturation [26], high fecundity, and spawning with a protracted reproductive season [12] also facilitate successful recruitment [27,28]. As a batch spawner, the round goby can spawn from four to six times a year from April to October at temperatures of 9–26 °C within a protracted spawning season at an interval of approximately 17–28 days [1,12,29,30]. At higher temperatures within the preferred temperature range, the interval between spawning batches may be shorter [12]. To allow for such frequent spawning bouts, the round goby must have a mechanism to produce mature eggs rapidly. The oocyte development of this species is continual, meaning all stages of egg development are present simultaneously in the ovary [31].

Data from different ecosystems confirm a range of strategies in the spawning behavior of the round goby. The age at maturity of round goby in the native range is between 3 and 4 years for males and 2–3 years for females, which can produce 328–5221 eggs per spawning event [29,32]. In contrast, specimens in introduced populations are described to mature earlier; for example, fish mature at an age of 1 to 2 years and produce only 198 eggs per batch in the Detroit River [27]. Sex ratio in stable native populations is nearly 1:1, while in introduced populations, the percentage of males can be considerably higher [31], with up to six males to one female [33]. The length of the reproductive season varies considerably depending on locality. In the Black and Caspian seas, spawning may begin as early as April and may continue to the end of June (Romania), July (Sea of Azov), or September (Varna, Bulgaria) [32]. In the Lower Rhine, the spawning intensity of the round goby is usually highest at the beginning of the reproductive season (April/May), but behavior varies strongly between years [34]. In the Baltic Sea area, the most advanced gonad stages were detected in April and July, indicating two peaks in spawning activity [31].

Over the past decades, much effort has been devoted to research on the ecological impacts of the round goby on native species and systems (e.g., trophic interactions, habitat use, interspecies competition). Other key research foci have been the rate and extent of invasion, life history traits of newly established populations, and management of the round goby [1,6,12,16,33]. Less research has been devoted to the reproductive biology, physiology, and behaviors of the round goby in southwestern Lake Michigan, and more studies are needed to better understand the complexities of its reproduction status [35]. In newly established populations in the Great Lakes, the pattern of oocyte development within a reproductive season remains poorly documented. Studies of gonad development have been done in native, as well as recently invaded European systems, where the round goby are often larger in size and have a higher age at maturity in comparison with round goby present in their newly established ranges [31,36]. Critical differences in the physiology of these globally dispersed populations likely produce differing reproductive characteristics, and thus, it is important to look at individual populations to determine spawning dynamics and effort.

The round goby has been present in the Great Lakes for more than four decades, and we have yet to fully understand the multifaceted reproductive habits of this influential species. In this study, we aimed to: (1) describe seasonal changes in GSI and oocyte development, (2) compare reproductive timing between two harbors with different thermal regimes, (3) quantify fecundity-length/weight relationships, and (4) evaluate the utility of GSI as a reproductive indicator using histology.

## 2. Methods and Materials

**Figure 1:**
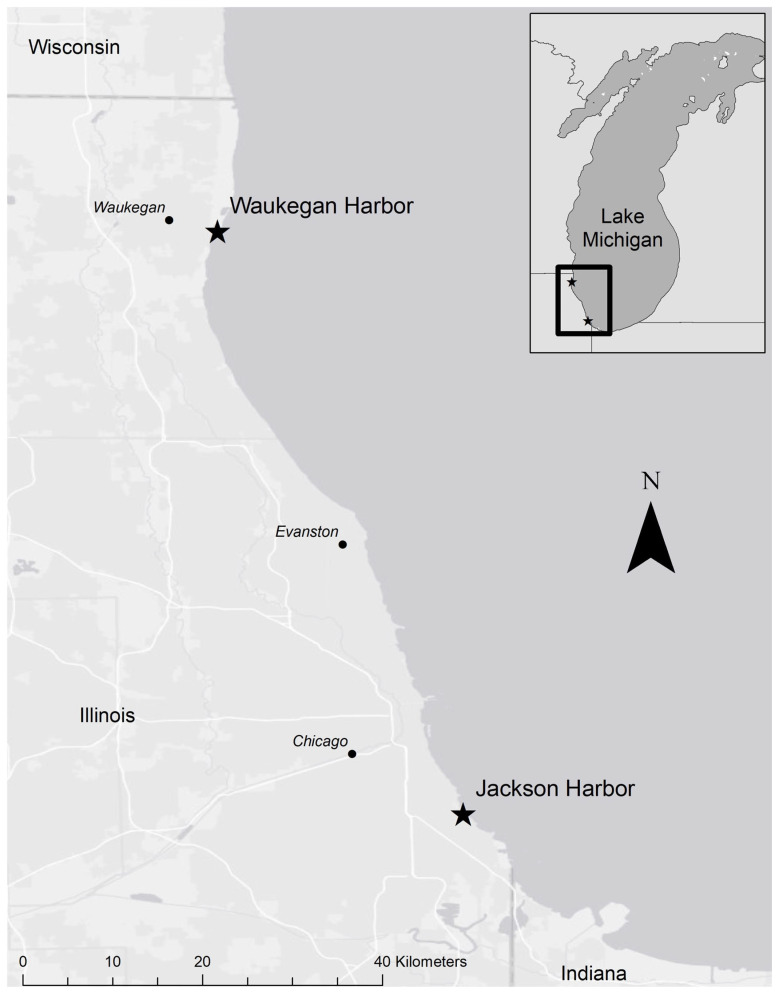
Map of collection sites in southwestern Lake Michigan: Waukegan Harbor and Jackson Harbor.

The round goby were collected with gillnet panels (mesh size 10–26 mm; length 30 m; height 1.5 m) from Jackson Harbor, IL, and Waukegan Harbor, IL (southwestern Lake Michigan, USA) from May to September (n = 552; Figure 1).

Samples described as early were collected between the 1st and 10th day of each month, samples described as middle were collected between the 11th and 20th day of each month, and samples designated as late were collected between the 21st and 30th or 31st day of each month. Our sampling scheme was designed to yield a representative sample of female round goby across a size range and throughout the reproductive season. Fish were anesthetized in overexposed aqueous solution (0.3 g dm^−3^) of MS-222 according to the United States federal law (The Humane Methods of Slaughter Act—HMSA), then total length, total weight, and ovary weight were recorded for all fish (to nearest 0.1 mm, 0.01 g, and 0.001 g, respectively). Reproductive condition was assessed using a gonadosomatic index (GSI) calculated as: total gonad mass/total body mass × 100 [37].

After extraction, the ovaries were preserved in modified Gilson’s fluid (100 mL 60% ethanol; 880 mL water; 15 mL 80% nitric acid; 18 mL glacial acetic acid; and 20 g mercuric chloride) for egg counts [38]. A subsample of fish from each site was used for egg counts, and mean egg lengths were recorded (n = 177). Eggs were counted by hand with the aid of a stereomicroscope after separation from the remaining ovarian tissue. Only ovaries with mature, yolked, and unovulated eggs were used to avoid underestimating fecundity due to spawned eggs or eggs lost during handling [39]. An additional subsample of females was prepared for histological analysis (n = 95). Females were taken from each sample throughout the season for equal representation of the reproductive season and then preserved and prepared for histology.

Temperature loggers (model HOBO UA-002-64, HOBO Data Loggers, Bourne, MA, USA) were placed at Waukegan Harbor (42°22′18″ N; 87°46′57″ W) and Jackson Harbor (41°59′72″ N; 87°54′39″ W) and recorded daily temperatures at a depth of 2 m during May–September.

### 2.1. Ovaries Histology

**Figure 2:**
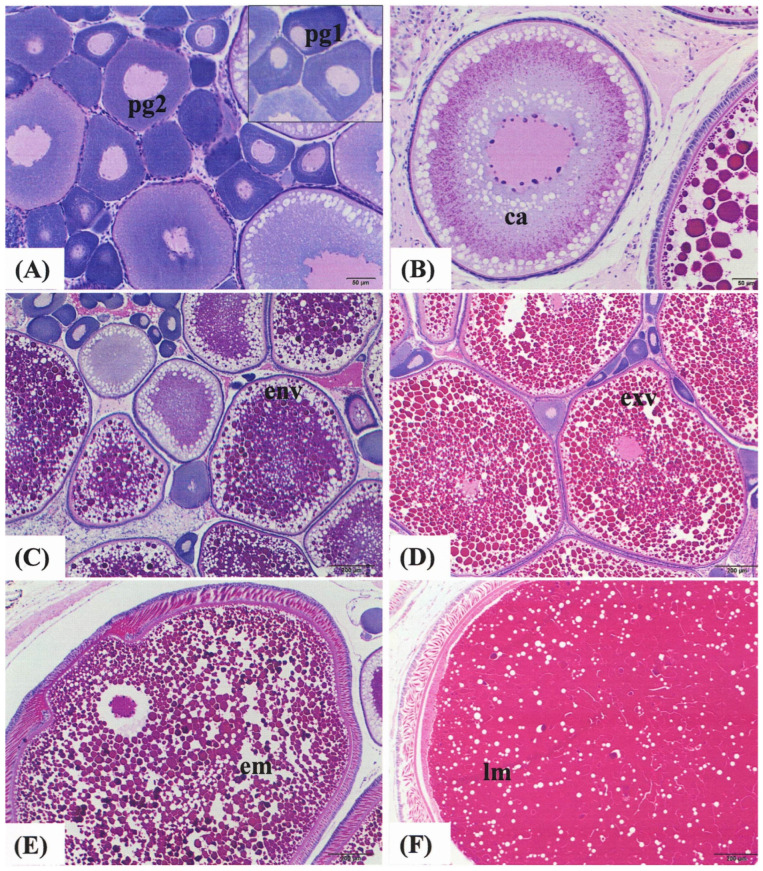
Oocytes of the round goby: (**A**) at stage 1; (**B**,**C**) at stage 2; (**D**,**E**) at stage 3; (**F**) at stage 4. ca—cortical alveolus phase; em—early maturation phase; env—endogenous vitellogenic; exv—exogenous vitellogenic; lm—late maturation phase; pg1—primary growth chromatin nucleolus phase; pg2—primary growth perinucleolus phase.

All histological samples were prepared by the Histology Lab in the College of Veterinary Medicine at the University of Illinois. Fish gonads used for histological sampling were processed into paraffin blocks using a Tissue-Tek VIP processor (Sakura Finetek, Inc., Torrance, CA, USA). This processor ran the samples through increasingly concentrated ethyl alcohol (70, 80%, 95%, 100%) to dehydrate them, after which the samples were immersed in xylene. The xylene was then replaced with melted paraffin to allow embedment in paraffin blocks. Paraffin blocks were then thinly sectioned with a rotary microtome (4–5 µm) and stained using the HE (haematoxylin–eosin) topographic method [40]. Histological analyses of cross-sections for the shape, size, and type of germ cells in the gonads were performed using an Olympus BX43 (Amtech Medical Ltd., Coral Springs, FL, USA) transmission light microscope and micro-image computer analysis cellSens Standard 1.7 software 7. Cells and cellular structures of the ovaries were described using the nomenclature of Brown-Peterson et al. [41]. Histological samples were examined by two independent readers with a compound microscope, and egg counts for each stage were recorded for each slice of the ovary (Figure 2). Three slices were read for each ovary, and an ovary average was calculated.

### 2.2. Statistical Analysis

All data were analyzed using SAS (version 9.2) and were examined for normality using Shapiro–Wilk tests and for equal variance using Brown–Forsythe tests. When normality and equal variance assumptions were not met, transformations were used. When transformations were ineffective, non-parametric tests were used: non-normal data were tested using Wilcoxon Rank Sum tests (two populations test) or Kruskal–Wallis tests (more than two populations test), and data with unequal variance were analyzed using Welch’s ANOVA. A one-way *t*-test was used to test the deviation of reproductive female GSI scores from the standard of 8.0 currently used to determine reproductive status. One-way ANOVA, followed by Tukey–Kramer post hoc tests, was used to test differences between proportions of oocytes in each stage over the reproductive season. Welch’s ANOVA was used to test differences between mean GSI scores of each oocyte stage. Pearson correlations were used to analyze the relationship between fecundity and total length and between fecundity and total weight. The Kruskal–Wallis test was used to test differences between egg counts for each period.

## 3. Results

### 3.1. Water Thermals at Research Sites

**Figure 3:**
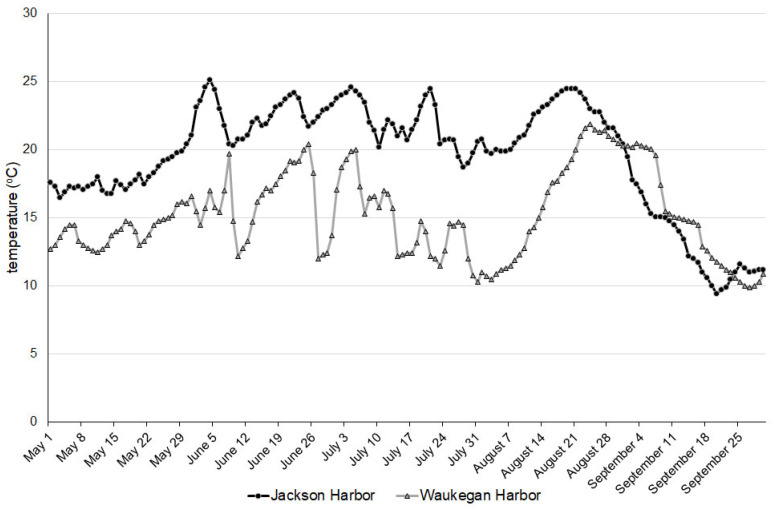
Temperature at sampling sites in Waukegan (42°22′18″ N; 87°46′57″ W) and Jackson Harbor (41°59′72″ N; 87°54′39″ W) during the analyzed season.

Generally, the water temperature at the two sites differed during the season. From the beginning of May to mid-August, lower values of the average daily temperature were recorded at Waukegan Harbor, where, especially in June, they fluctuated significantly. The water temperature within Jackson Harbor was more stable and fluctuated slightly during the analyzed season (Figure 3).

### 3.2. Patterns Within Reproductive Season

**Figure 4:**
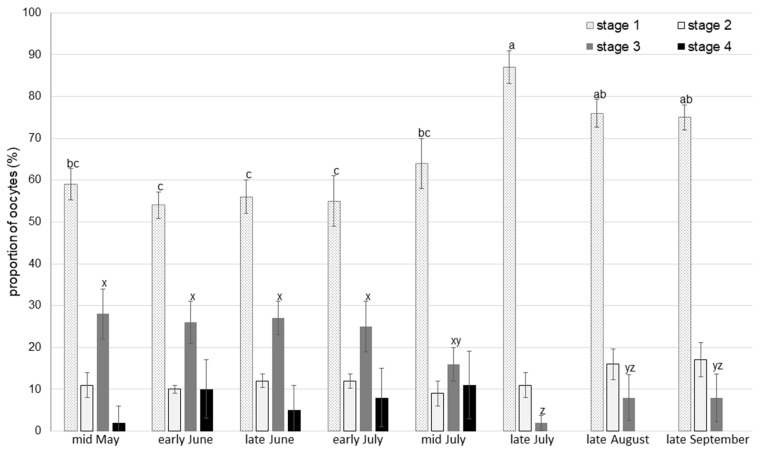
Proportion of different stages of oocytes in round goby ovaries during the analyzed season. Values (mean ± SD) with different superscripts are significantly different (*p* < 0.0001) when compared within a given stage of oocyte development (a, b, c—stage 1), (x, y, z—stage 3).

Significant differences in the proportion of oocytes at each stage over time (sampling period) were found for stage 1 (n = 95, F = 7.98, *p* < 0.0001) and stage 3 (n = 84, F = 7.37, *p* < 0.0001) (Figure 4). No significant differences were detected for stage 2 (n = 94, F = 0.87, *p* = 0.5228) and stage 4 (n = 19, χ^2^ = 9.305, *p* = 0.1571). Significantly higher proportions of immature (stage 1) oocytes were present after peak spawning season, and significantly higher proportions of mature (stage 3) oocytes were present during peak spawning activity. Although not statistically significant, oocytes in the ready-to-spawn stage (stage 4) were only present early June through mid July, which is consistent with peak spawning activity in this population.

### 3.3. Patterns Across Sites and Determination of Females’ Reproductive Status

**Figure 5:**
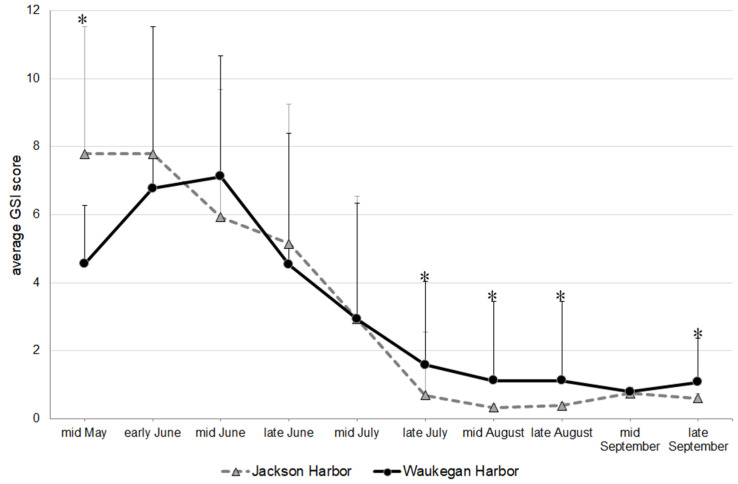
Changes in mean values of round goby GSI over time between Waukegan and Jackson Harbors. Asterisks indicate significant differences between sampling sites during the analysis season (*p* < 0.0001).

**Figure 6:**
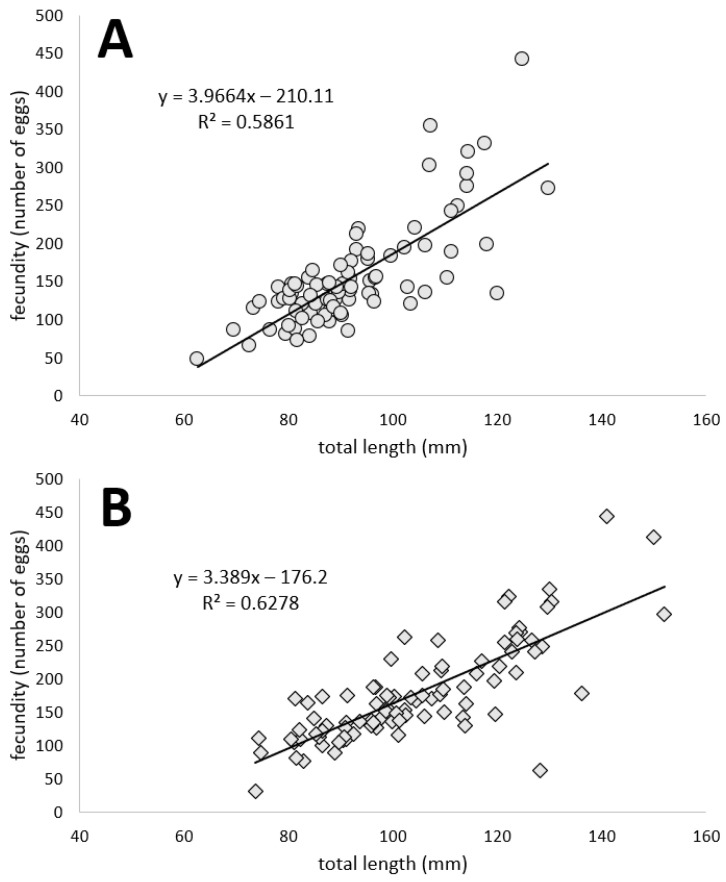
Relationship between round goby (*Neogobius melanostomus*) female length and absolute fecundity from: (**A**)—Jackson Harbor (N = 84) and (**B**)—Waukegan Harbor (N = 91).

**Figure 7:**
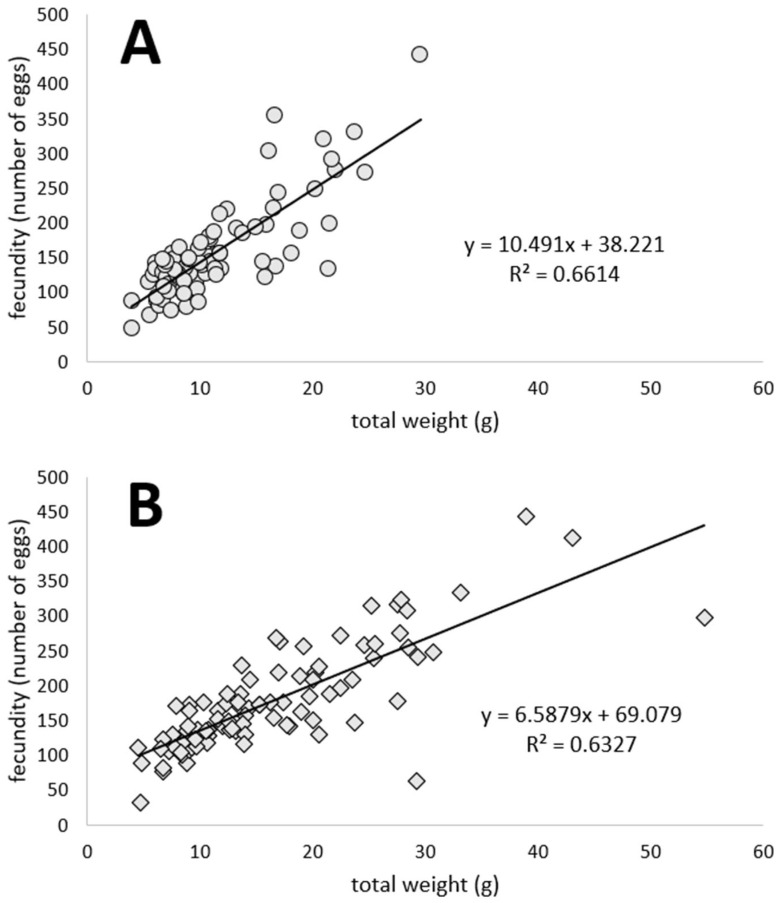
Relationship between round goby (*Neogobius melanostomus*) female weight and absolute fecundity from: (**A**)—Jackson Harbor (N = 84) and (**B**)—Waukegan Harbor (N = 91).

Significant differences were found for mean GSI scores on the tails of the reproductive season between Jackson Harbor and Waukegan Harbor (*p* < 0.01; Figure 5). Mean GSI differed significantly between sites in mid-May (F = 9.33, *p* = 0.0104), late July (Z = 5.1414, *p* < 0.0001), mid-August (Z = −5.4026, *p* < 0.0001), late August (Z = −6.3273, *p* < 0.0001), and late September (Z = 3.8839, *p* < 0.0001). Fecundity in the range between 31 and 443 eggs (168 ± 72 mean ± SD) was significantly positively related to female total length at both sites (Jackson Harbor, *p* < 0.0001, R^2^ = 0.5861, and Waukegan Harbor, *p* < 0.0001, R^2^ = 0.6278), respectively (Figure 6). Similarly, there was a relationship between fecundity and total mass of females (Jackson Harbor, *p* < 0.0001, R^2^ = 0.6614) and Waukegan Harbor (*p* < 0.0001, R^2^ = 0.6327) (Figure 7).

**Figure 8:**
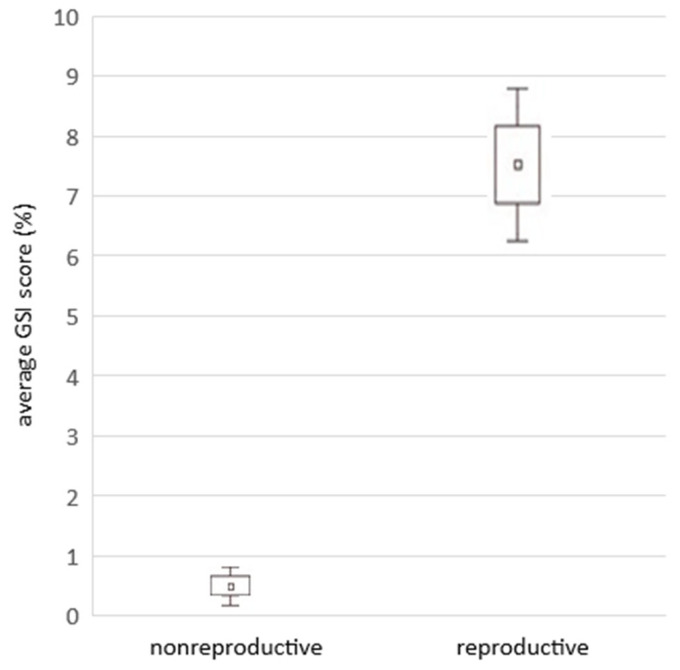
Mean value of GSI in round goby females with nonreproductive and reproductive status from Jackson Harbor and Waukegan Harbor.

**Figure 9:**
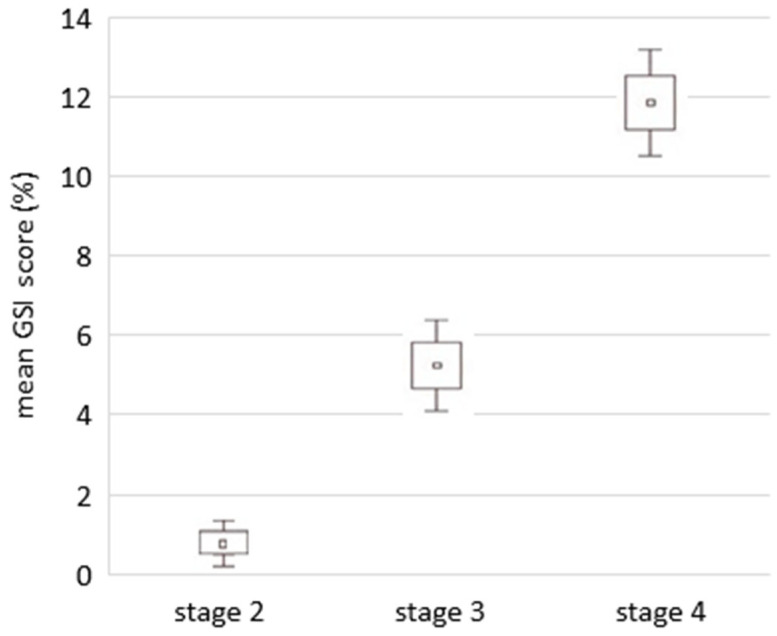
Relationship between the mean GSI score and the dominant stage of oocyte development of the round goby females caught at Waukegan and Jackson Harbors.

In Waukegan Harbor, where pre- and post-spawning peak data were captured, approximately 50% of the females captured were reproductive. Mean egg count per sample period in both harbors did not differ significantly throughout the season (χ^2^ = 2.8171, *p* = 0.8314). Mean GSI score of reproductive females was significantly greater than the mean GSI of nonreproductive fish (*p* < 0.0001, mean ± SD reproductive GSI = 7.5 ± 0.65%, mean ± SD nonreproductive GSI = 0.5 ± 0.1%, Figure 8). Mean GSI for each oocyte stage differed significantly from each other (*p* < 0.0001, Figure 9).

## 4. Discussion

Timing of spawning peaks differed significantly between two relatively proximate harbors. This is likely due to slightly different thermal regimes and thus offset timing of optimal spawning temperatures. Although the seasonal temperature trends are similar in the two locations, the timing of the threshold temperature required for spawning is predicted to differ between them due to the higher temperatures noted in Jackson Harbor. As temperature has been cited as a cue for spawning in many fish species, we anticipated that water temperature would be a driver in the timing of spawning peaks for our sites. In Waukegan Harbor, data were captured for both pre- and post-spawning peaks. Our data show that the spawning peak was characterized by approximately 50% of the females captured being reproductively mature, with the highest proportion of oocytes at stage 4 in the gonads. The largely unchanged mean egg counts over time at both sites are indicative of continual oocyte development and the maturation of several batches of oocytes during the prolonged reproductive season.

The round goby has a protracted reproductive season and thus spawns multiple times throughout that period, but small portions of our samples would still include reproductive females regardless of month. The detection of stage 4 oocytes in females during the first two months of the study showed that they were capable of extended spawning. In addition, the histological development in the adult stage of the gonads (stages 3 and 4) showed the appearance of various types of oocytes at the immature stages, such as previtellogenic and primary growth oocytes. Therefore, after the mature oocytes were released, the lower-grade oocytes continued to develop and continued the cycle. This confirms that the reproductive form of the round goby in southwestern Lake Michigan was spawning many times during the spawning season.

Our data documented highest immature oocyte proportions occurred at the end of the spawning season, and the highest mature oocyte proportions at the peak of spawning activity. These changes in the proportion of egg stages were reflected in mean GSI scores per period. No significant differences in stages 2 and 4 throughout the season were noted. When stage 4 (the most mature oocytes) are present in the ovary, they take up much more space and thus counts for this stage are consistently lower, leading to an overall lower number of stage 4 eggs counted. Conversely, there are consistently high counts for stage 2 oocytes, and thus, we predict that the consistency of the proportion of stage 2 oocytes present in the ovaries reflects the continual oocyte development characteristic of this species. Continual oocyte development leads to multiple stages of oocytes present in the ovaries simultaneously, and hence, the inverse relationship we see with stage 1 and stage 3 oocyte proportions is likely reflective of immature oocytes replacing mature oocytes as the season progresses.

The estimated absolute fecundity of the round goby females from southwestern Lake Michigan is lower than that from the native round goby regions, where it ranges from 328 to 5221 eggs [29]. Fecundity of the round goby is often positively correlated to both size and mass in fishes [27,31,42,43]. As expected, our results confirm a significant correlation between egg number and body size of the round goby females inhabiting southwestern Lake Michigan. The linear relation of total length or total weight and fecundity of fish corresponded to data reported by McInnis and Corkum [27]. Individual fecundity of the round gobies collected from the upper Detroit River ranged from 84 to 606 eggs and was significantly correlated with fish size (R^2^ = 0.76, *p* < 0.001). A similar range of fecundity, between 86 and 591 eggs (mean value 177 eggs), was estimated in female round gobies in six Pennsylvania tributary streams of Lake Erie, and fecundity was closely related to the length of gravid females (R^2^ = 0.87, *p* < 0.0001) [42]. However, the absolute (total) fecundity of females caught in three tributaries of the Lower Danube River was higher and varied from 159 to 773 oocytes (mean of 360 oocytes). The batch fecundity was from 32 to 507 oocytes (mean of 162), and relative fecundity ranged from 55 to 134 oocytes (mean of 94 oocytes). There was a linear relationship between absolute fecundity and total length (R^2^ = 0.63) with a SE of 94.02 for the intercept and 14.42 for the slope of total length and total weight (R^2^ = 0.75) [43]. In the Gulf of Gdańsk, absolute fecundity ranged widely from 94 to 2190 eggs (mean 645, SD 423) at a female of the round goby length of 59–187 mm [31] and there was a power relationship between absolute fecundity and total length (r = 0.7206, *p* < 0.005) and a significant correlation between absolute fecundity and female body weight (r = 0.7913, *p* < 0.005). Wandzel [44] reported similar relationships, while the fish from the Gulf of Gdańsk during the same period noted a higher absolute fecundity range of 89 to 3824 eggs (mean 1739) at a mean female length of 74–166 mm.

GSI is widely regarded to measure sexual maturity in relation to the ovaries’ development, but during the histological analysis of oocyte hydration and description of final maturation in the round goby collected in the Gulf of Gdańsk (Baltic Sea), GSI was an inaccurate estimator of oocyte maturation [45]. In this study, GSI values were below 8% in stages 1 (early germinal vesicle migration), 4 (final hydration), and 5 (ovulation), while histological analysis indicated that all females were spawning-capable. In multiple-spawning fish, GSI seems to be a less reliable estimator of fish maturity, which does not provide an accurate estimation of ovarian maturity or differentiation between females in a spawning-capable stage and those in a post-spawning stage. Nonetheless, if significant gonad growth occurs at distinct developmental stages (e.g., vitellogenesis), GSI cut-off scores (i.e., designating those above the score as “reproductive” or “spawning-capable”) may be useful metrics for sorting fish into gross reproductive categories [46].

For female round gobies in the Upper Danube River, GSI levels ranged from 0.01% to 19.62%. While the highest mean GSI values were observed in April of the years 2022–2023, the lowest levels were found in August 2022 and September 2023 [47], similar to our data. However, in the Lower Danube (Bulgaria), peaks in the GSI were observed in February and May [43], and in the Trent–Severn Waterway (Canada), where the highest values were found after winter [48]. It is worth noting that in their native range, round gobies’ spawning season was observed between March and May, with a maximum value of GSI also in April and a minimum in August [49]. Similar to the native range and to data collected in the Kiel Canal and the Lower Danube, where the lowest GSI was reported in September [9] and from August to November [43], the lowest GSI levels in this study were found in August and September. Furthermore, a current and widely used standard procedure for determining reproductive status in female round goby is to calculate gonadosomatic index scores and regard any females with GSI scores greater than 8% to be mature and those with scores less than 8% to be immature [50,51]. It is important to verify this standard as a useful and relatively inexpensive tool for managers to determine the status and reproductive potential of the round goby populations they manage.

## 5. Conclusions

This study evaluated reproductive status and spawning effort of round goby females in southwestern Lake Michigan. The timing of spawning peaks differed between two relatively proximate sites, i.e., Jackson Harbor (JH) and Waukegan Harbor (WH), and occurred at 14–17 °C. Peak spawning effort, as determined by GSI score, correlated with the highest proportions of mature oocytes in the ovaries, thus confirming the usefulness of GSI as a reliable indicator of spawning activity. Our data showed that mature, “ready-to-spawn” female GSI scores were significantly higher than eight, and the GSI scores of non-reproductive and reproductive females differed significantly. Fecundity was positively correlated with the total length and total weight of individual females.

Research on body size, fecundity, and oocyte development will provide valuable information not only for a more complete understanding of the basics of round goby reproductive biology in the Great Lakes but also for comparative purposes between native systems and newly invaded European systems. Differences in populations along various invasion fronts and native systems may provide useful insights for devising management and prevention strategies in the future.

## Data Availability

The original contributions presented in this study are included in the article. Further inquiries can be directed to the corresponding author.

## References

[1] Kornis M.S., Mercado-Silva N., Vander Zanden M.J. (2012). Twenty years of invasion: A review of round goby *Neogobius melanostomus* biology, spread and ecological implications. J. Fish Biol..

[2] Sapota M.R., Skora K.E. (2005). Spread of alien (non-indigenous) fish species *Neogobius melanostomus* in the Gulf of Gdansk (south Baltic). Biol. Invasions.

[3] Ojaveer H. (2006). The round goby *Neogobius melanostomus* is colonizing the NE Baltic Sea. Aquat. Invasions.

[4] Bjȍrklund M., Almqvist G. (2010). Rapid spatial genetic differentiation in an invasive species, the round goby *Neogobius melanostomus* in the Baltic Sea. Biol. Invasions.

[5] Sokołowska E., Fey D.P. (2011). Age and growth of the round goby *Neogobius melanostomus* in the Gulf of Gdańsk several years after invasion. Is the Baltic Sea a new Promised Land?. J. Fish Biol..

[6] Verliin A., Kesler M., Svirgsden R., Taal I., Saks L., Rohtla M., Hubel K., Eschbaum R., Vetemaa M., Saat T. (2017). Invasion of round goby to the temperate salmonid streams in the Baltic Sea. Ichthyol. Res..

[7] Borcherding J., Staas S., Krűger S., Ondračková M., Šlapanský L., Jurajda P. (2011). Non-native Gobiid species in the lower River Rhine (Germany): Recent range extensions and densities. J. Appl. Ichthyol..

[8] Roche K.F., Janáč M., Jurajda P. (2013). A review of Gobiid expansion along the Danube-Rhine corridor—Geopolitical change as a driver for invasion. Knowl. Manag. Aquat. Ecosyst..

[9] Hempel M., Magath V., Neukamm R., Thiel R. (2019). Feeding ecology, growth and reproductive biology of round goby *Neogobius melanostomus* (Pallas, 1814) in the brackish Kiel Canal. Mar. Biodivers..

[10] Apostolou A., Velkov B., Green L. (2022). The first record of the invasive round goby *Neogobius melanostomus* in the Aegean Basin, Bulgaria. J. Appl. Ichthyol..

[11] Jude D.J., Reider R.H., Smith G.R. (1992). Establishment of Gobiidae in the Great Lakes basin. Can. J. Fish. Aquat. Sci..

[12] Charlebois P.M., Marsden J.E., Goettel R.G., Wolfe R.K., Jude D.J., Rudnicka S. (1997). The Round Goby, Neogobius melanostomus (Pallas), a Review of European and North American Literature.

[13] Jude D.J., Janssen J., Crawford G., Munawar M., Edsal T., Leach J. (1995). Ecology, distribution and impact of the newly introduced round and tubenose gobies on the biota of the St. Clair and Detroit Rivers. The Lake Huron Ecosystem: Ecology, Fisheries and Management.

[14] Brown J.E., Stepien C.A. (2009). Invasion genetics of the Eurasian round goby in North America: Tracing sources and spread patterns. Mol. Ecol..

[15] Hayden T.A., Miner J.G. (2009). Rapid dispersal and establishment of a benthic Ponto-Caspian goby in Lake Erie: Diel vertical migration of early juvenile round goby. Biol. Invasions.

[16] Charlebois P.M., Corkum L.D., Jude D.J., Knight C. (2001). The round goby (*Neogobius melanostomus*) invasion: Current research and future needs. J. Gt. Lakes Res..

[17] Dillon A.K., Stepien C.A. (2001). Genetic and biogeographic relationships of the invasive round (*Neogobius melanostomus*) and tubenose (*Proterorhinus marmoratus*) gobies in the Great Lakes versus Eurasian populations. J. Gt. Lakes Res..

[18] Rawlings C.C., Campbell S.E., Mandrak N.E. (2021). Body shape variation in round goby *Neogobius melanostomus* in the Laurentian Great Lakes basin. Environ. Biol. Fishes.

[19] Irons K.S., McClelland M.A., Pegg M.A. (2006). Expansion of round goby in the Illinois waterway. Am. Midl. Nat..

[20] Campbell T.B., Tiegs S.D. (2012). Factors governing the distribution and fish-community associations of the round goby in Michigan tributaries of the Laurentian Great Lakes. J. Gt. Lakes Res..

[21] Kornis M.S., Vander Zanden M.J. (2010). Forecasting the distribution of the invasive round goby (*Neogobius melanostomus*) in Wisconsin tributaries to Lake Michigan. Can. J. Fish. Aquat. Sci..

[22] Kornis M.S., Weidel B.C., Vander Zanden M.J. (2017). Divergent life histories of invasive round gobies (*Neogobius melanostomus*) in Lake Michigan and its tributaries. Ecol. Freshw. Fish.

[23] Diggins T.P., Kaur J., Chakraborti R.K., DePinto J.V. (2002). Diet choice by the exotic round goby (*Neogobius melanostomus*) as influenced by prey motility and environmental complexity. J. Gt. Lakes Res..

[24] Copp G.H., Kováč V., Zweimüller I., Dias A., Nascimento M., Balážová M. (2008). Preliminary study of dietary interactions between invading Ponto-Caspian gobies and some native fish species in the River Danube near Bratislava (Slovakia). Aquat. Invasions.

[25] Polačik M., Janáč M., Jurajda P., Adámek Z., Ondračková M., Trichkova T., Vassilev M. (2009). Invasive gobies in the Danube: Invasion success facilitated by availability and selection of superior food resources. Ecol. Freshw. Fish.

[26] L’avrinčíkova M., Kováč V. (2007). Invasive round goby *Neogobius melanostomus* from the Danube mature at small size. J. Appl. Ichthyol..

[27] MacInnis A.J., Corkum L.D. (2000). Fecundity and reproductive season of the round goby *Neogobius melanostomus* in the upper Detroit River. Trans. Am. Fish. Soc..

[28] Sapota M.R. (2004). The round goby (*Neogobius melanostomus*) in the Gulf of Gdansk—A species introduction into the Baltic Sea. Hydrobiologia.

[29] Kovtun I.F. (1977). On the fecundity of the round goby, *Gobius melanostomus*, from the Sea of Azov. J. Ichthyol..

[30] Corkum L.D., MacInnis A.J., Wickett R.G. (1998). Reproductive habits of round gobies. Gt. Lakes Res. Rev..

[31] Tomczak M.T., Sapota M.R. (2006). The fecundity and gonad development cycle of the round goby (*Neogobius melanostomus* Pallas 1811) from the Gulf of Gdansk. Oceanol. Hydrobiol. St..

[32] Miller P.J., Whitehead P.J.P., Bauchot M.L., Hureau J.C., Neilson J., Tortonese E. (1986). Gobiidae. Fishes of the Northeastern Atlantic and the Mediterranean.

[33] Corkum L.D., Sapota M.R., Skora K.E. (2004). The round goby, *Neogobius melanostomus*, a fish invader on both sides of the Atlantic Ocean. Biol. Invasions.

[34] Gertzen S., Fidler A., Kreische F., Kwabek L., Schwamborn V., Borcherding J. (2016). Reproductive strategies of three invasive Gobiidae co-occurring in the Lower Rhine (Germany). Limnol. Ecol. Manag. Inland Waters.

[35] Marentette J.R., Fitzpatrick J.L., Berger R.G., Balshine S. (2009). Multiple male reproductive morphs in the invasive round goby (*Apollonia melanostoma*). J. Gt. Lakes Res..

[36] Kulikova N.I. (1985). The effect of chorionic gonadotropin on growth and maturation of the oocytes of the round goby, *Neogobius melanostomus*. J. Ichthyol..

[37] Crim L.W., Glebe B.D., Schreck C.B., Moyle P.B. (1990). Reproduction. Methods for Fish Biology.

[38] Snyder D.E., Nielsen L.A., Johnson D.L. (1983). Fish eggs and larvae. Fisheries Techniques.

[39] Hôrková-Žitňanová K., Švolíková K., Haruštiaková D., Kováč V. (2021). A new approach to evaluate reproductive traits in batch-spawning fishes of indeterminate fecundity and asynchronous oocyte maturation. Rev. Fish. Sci. Aquac..

[40] Zawistowski S. (1986). Histological Techniques, Histology and the Bases of Histopathology.

[41] Brown-Peterson N.J., Wyanski D.M., Saborido-Rey F., Macewicz B.J., Lowerre-Barbieri S.K. (2011). A standardized terminology for describing reproductive development in fishes. Mar. Coast Fish. Dyn. Manag. Ecosyst. Sci..

[42] Phillips E.C., Washek M.E., Hertel A.W., Niebel B.M. (2003). The round goby (*Neogobius melanostomus*) in Pennsylvania tributary streams of Lake Erie. J. Gt. Lakes Res..

[43] Dashinov D.D., Uzunova E.P. (2021). Reproductive biology of pioneer round gobies (*Neogobius melanostomus* Pallas, 1814) at the edge of their invasion front in three small rivers (Lower Danube Basin, Bulgaria). J. Vertebr. Biol..

[44] Wandzel T. (2000). The fecundity and reproduction of round goby *Neogobius melanostomus* (Pallas, 1811) in the Puck Bay (Baltic Sea). Bull. Sea Fish. Inst..

[45] Kalamarz-Kubiak H., Guellard T. (2019). Oocyte hydration in round goby *Neogobius melanostomus* from the Gulf of Gdańsk: Another invasive strategy?. Oceanologia.

[46] Zeyl J.N., Love O.P., Higgs D.M. (2014). Evaluating gonadosomatic index as an estimator of reproductive condition in the invasive round goby, *Neogobius melanostomus*. J. Gt. Lakes Res..

[47] Klarl M., Pander J., Geist J. (2024). Characterization of the reproductive strategy of invasive round goby (*Neogobius melanostomus*) in the Upper Danube River. Ecol. Evol..

[48] Houston B.E., Rooke A.C., Brownscombe J.W., Fox M.G. (2014). Overwinter survival, energy storage and reproductive allocation in the invasive round goby (*Neogobius melanostomus*) from a river system. Ecol. Fresh. Fish.

[49] Aydin M. (2021). Age, growth and reproduction of *Neogobius melanostomus* (Pallas 1814) (Perciformes: Gobiidae) in the southern Black Sea. Mar. Sci. Technol. Bull..

[50] MacInnis A.J. (1997). Aspects of the Life History of the Round Goby, *Neogobius melanostomus* (Perciformes: Gobiidae), in the Detroit River. Master’s Thesis.

[51] Gammon D.B., Li W., Scott A.P., Zielinski B.S., Corkum L.D. (2005). Behavioural responses of female *Neogobius melanostomus* to odours of conspecifics. J. Fish Biol..

